# The Effect of Inhaled Corticosteroids on Pneumonia Risk in Patients With COPD-Bronchiectasis Overlap: A UK Population-Based Case-Control Study

**DOI:** 10.1016/j.chest.2023.06.007

**Published:** 2023-06-17

**Authors:** Andrew I. Ritchie, Aran Singanayagam, Sebastian Mitchell, Jadwiga A. Wedzicha, Anand Shah, Chloë I. Bloom

**Affiliations:** a National Heart and Lung Institute, Imperial College London, London, England; b Department of Infectious Disease, Imperial College London, London, England; c Department of Infectious Disease Epidemiology, MRC Centre for Global Infectious Disease Analysis, Imperial College London, London, England; d Royal Brompton Hospital, Guys and St. Thomas’ NHS Foundation Trust, London, England; e Imperial College Healthcare NHS Trust, London, England

**Keywords:** bronchiectasis, COPD, inhaled corticosteroid, pneumonia

## Abstract

**Background:**

Inhaled corticosteroids (ICS) increase the risk of pneumonia in COPD and commonly are used in patients with COPD-bronchiectasis overlap.

**Research Question:**

Is the risk of pneumonia associated with ICS further heightened in COPD-bronchiectasis?

**Study Design and Methods:**

Electronic health care records (from 2004-2019) were used to obtain a cohort of patients with COPD and a nested case-control group (age and sex matched 1:4). Analyses were conducted to determine the risk of hospitalization for pneumonia in COPD associated with ICS use in those with bronchiectasis. Findings were confirmed by several sensitivity analyses. Additionally, a smaller nested case-control group containing only patients with COPD-bronchiectasis overlap and those with recent blood eosinophil counts (BECs) was used to determine any association with BEC.

**Results:**

Three hundred sixteen thousand six hundred sixty-three patients were eligible for the COPD cohort; bronchiectasis significantly increased the risk of pneumonia (adjusted hazard ratio, 1.24; 95% CI, 1.15-1.33). In the first nested case-control group of 84,316 patients with COPD, ICS was found to increase the odds of pneumonia (adjusted OR [AOR], 1.26; 95% CI, 1.19-1.32) only if used in the previous 180 days. However, bronchiectasis was a significant modifier such that ICS use did not augment further the already elevated bronchiectasis-associated pneumonia risk (COPD-bronchiectasis: AOR, 1.01; 95% CI, 0.8-1.28; no bronchiectasis: AOR, 1.27; 95% CI, 1.20-1.34). Several sensitivity analyses and a second smaller nested case-control group confirmed these findings. Finally, we found that BEC modified the ICS-associated pneumonia risk in COPD-bronchiectasis overlap, where lower BEC was associated significantly with pneumonia (BEC ≤ 3 × 10^9^/L: AOR, 1.56; 95% CI, 1.05-2.31; BEC > 3 × 10^9^/L: AOR, 0.89; 95% CI, 0.53-1.24).

**Interpretation:**

ICS use does not augment further the already increased risk of hospitalization for pneumonia associated with concomitant bronchiectasis in patients with COPD.

FOR EDITORIAL COMMENT, SEE PAGE 809

**Take-home Points**

**Study Question:** Is the use of inhaled corticosteroids (ICS) associated with an enhanced risk of pneumonia in people with COPD-bronchiectasis overlap as compared with those with COPD alone?

**Results:** ICS did not augment the already elevated pneumonia risk in those with COPD-bronchiectasis overlap, but this seemed to be found only in those with an elevated eosinophil count.

**Interpretation:** An elevated blood eosinophil count seems to protect patients with COPD from any additional ICS-associated increased pneumonia risk that might be expected with concomitant bronchiectasis.

Inhaled corticosteroids (ICS) are prescribed commonly for COPD and currently are recommended to reduce the risk of exacerbations in those with severe COPD.^1^, ^2^, ^3^ Their usefulness is debated because they have little impact on lung function; are not effective in all patients^4^^,^^5^; and increase the risk of pneumonia, *Pseudomonas* outgrowth, and mycobacterial infections.^6^, ^7^, ^8^, ^9^

ICS prescribing also is cautioned in those with COPD-bronchiectasis overlap.^10^ Underdiagnosis of bronchiectasis is common, but reports estimate that the burden of bronchiectasis varies from 4% of the 2,164 patients with COPD in the Evaluation of COPD Longitudinally to Identify Predictive Surrogate End-points (ECLIPSE) cohort^11^ to 58% in a smaller cohort of 92 patients with COPD.^12^ COPD-bronchiectasis overlap is associated with more severe COPD,^10^ a higher risk of community-acquired pneumonia (CAP),^13^ prolonged hospitalization for COPD exacerbations,^14^ and higher mortality.^15^ The characteristic dysregulated immunity and impaired airway clearance results in mucus accumulation and increased susceptibility to acute and persistent lung infections. Therefore, it may be anticipated that ICS further dampens host-defense mechanisms, leading to an even greater risk of pneumonia.

No studies of a COPD-bronchiectasis overlap cohort have compared ICS use directly with no ICS use, nor have they addressed this question using real-world data, which typically have the advantages of larger sample size and a wealth of prescribing data. Interestingly, a US study of patients with primary bronchiectasis used Medicare data to compare the effect of macrolide therapy with that of ICS. The study investigated respiratory infection in general and found that patients using ICS—as compared with those using macrolide antibiotics—were at an increased risk of all respiratory infections requiring hospitalization, including respiratory viral infections, bacterial infections, and bronchiectasis exacerbations.^16^ However, because the effect of ICS was in comparison with the effect of only the antibiotic, it is difficult to extrapolate conclusions regarding ICS-associated risks directly from that study. Furthermore, the study population was patients with primary bronchiectasis, but patients with COPD-bronchiectasis overlap may demonstrate a different ICS-associated infection risk profile. Martinez-Garcia et al,^17^ in a study of 201 selected patients with COPD, of whom just more than one-half had bronchiectasis, found ICS use and bronchiectasis both to be independent risk factors for pneumonia; however, they did not assess the interaction of the two risk factors.

The latest European Respiratory Society adult bronchiectasis guidelines acknowledged this paucity of evidence in COPD-bronchiectasis overlap and conclude that “in the absence of definitive data, the presence of bronchiectasis should not lead to a decision to withdraw ICS from patients with established COPD.”^18^ Herein, we conducted a study that leveraged a nationwide data source enriched with prescription data to investigate if ICS use—compared with no ICS use—further augments the risk of hospitalization for pneumonia in those with an already elevated pneumonia risk because of the presence of COPD-bronchiectasis overlap.

## Study Design and Methods

### Study Design and Data Source

The Clinical Practice Research Datalink Aurum is a database of pseudoanonymized UK primary care electronic health care records that includes approximately 20% of the UK population and has been shown to be nationally representative.^19^ This represents one of the largest, extensively validated longitudinal health care databases worldwide.^20^ Clinical Practice Research Datalink data were linked individually to Hospital Episode Statistics data (hospital admissions for England), socioeconomic data (Index of Multiple Deprivation; see Covariates section) and Office of National Statistics mortality data.

### Study Population and Design

**Figure 1:**
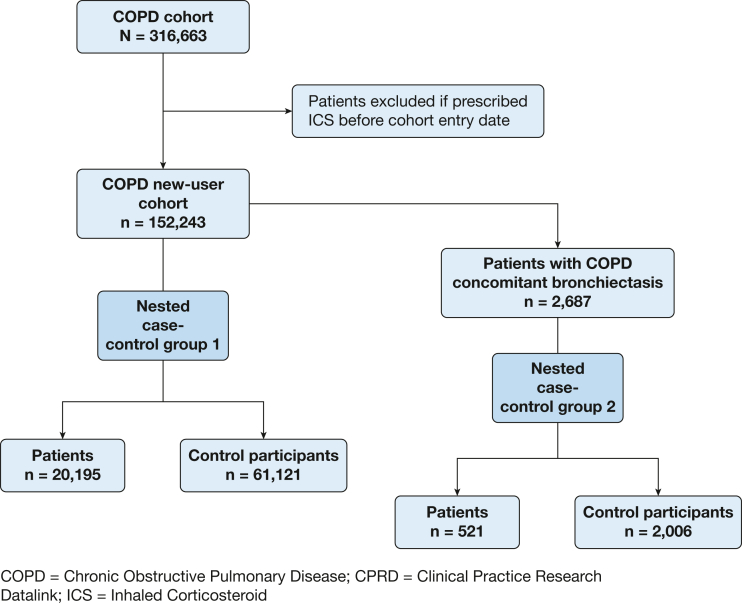
Flow diagram outlining the selection of the study cohort and the two nested case-control study populations. ICS = inhaled corticosteroids.

We identified patients with COPD using validated Read codes.^21^ Patients’ cohort entry date occurred on the latest date of the COPD diagnosis; January 1, 2004; 1 year after the general practitioner practice registration; or their 35th birthday. Patients were censored on the minimum date of their transfer out of their general practitioner practice, death, or January 1, 2019. Patients with cystic fibrosis were excluded. To create an incident ICS new-user cohort, patients who were prescribed ICS before cohort entry were excluded. A new-user design mitigates bias because it enforces appropriate temporal ordering of measurements of confounders, treatment, and outcome. Two nested case-control groups were derived from the new-user cohort. Nested case-control group 1 included all possible patients with COPD and matched control participants. As a sensitivity analysis, a second, much smaller nested case-control group 2 was drawn including only patients and control participants who had COPD-bronchiectasis overlap and a recent blood eosinophil count (BEC) (Fig 1). Because of the nature of case-control groups—only selecting patients from the cohort and then those control participants who match by the criteria set—these greatly reduce the number of patients as compared with the original cohort from which the case-control group was nested.

### Patient Identification

Patients who attended hospital with CAP, identified using International Classification of Diseases, Tenth Revision, codes J15 (pneumonia, organism unspecified) and J18 (bacterial pneumonia), were enrolled. We set the date of the hospital admission for pneumonia as the index date. Each patient was matched on the index date using incidence density sampling, randomly selecting control participants who matched on year of birth and sex from the pool of available control participants. Patients were matched preferably to four control participants; when four were not available, patients were matched to three control participants.

### Exposure Measurement

We examined all ICS prescription records in the year before the index date. ICS use was categorized as (1) a binary variable (use or no use), (2) time since the most recent prescription (current, ≤ 30 days; recent, 31-90 days; past, 91-180 days; and remote, > 180 days), (3) total number of ICS prescriptions, and (4) type of ICS (beclomethasone, fluticasone, budesonide, ciclesonide, or mometasone).

### Covariates

All covariates in the cohort were ascertained at entry, whereas covariates in the nested case-control group were ascertained on the index date. BMI was measured using kilograms per square meter. A history of bronchiectasis (diagnosed before cohort entry, but could have been before or after COPD diagnosis), as well as CAP, lower respiratory tract infections, depression, anxiety, heart failure, cardiovascular disease, diabetes, asthma, cerebrovascular disease, chronic renal disease, lung cancer, and interstitial lung disease (ILD), was recorded using appropriate Read codes reviewed by two physicians (A. I. R. and C. I. B.) at the time of entry into the cohort (for the cohort analysis) and were measured again at 1 year before the index date (for the nested case-control group). The covariate Index of Multiple Deprivation is the official measure of social deprivation in the United Kingdom and is based on seven domains: income, employment, education, health, crime, housing, and living environment deprivation. Prescription records were used to identify past vaccinations, pneumococcal (ever), and influenza (in the past year). Indicators of COPD severity were FEV_1_ % predicted, Medical Research Council dyspnea score, COPD exacerbation events requiring hospitalization, and the number of short courses (≤ 7 days) of oral corticosteroids (OCS) to treat acute COPD exacerbations in the year before cohort entry or index date. Absolute BECs, using the maximum value during the past 2 years, were categorized using the cutoff of 0.3 × 10^9^/L as either high or normal.^22^ Antibiotic use was defined as any oral antibiotic prescription used for lower respiratory tract infections (penicillins, fluoroquinolones, cephalosporins, and macrolides) and separately, specifically macrolides.

### Statistical Analysis

The main analysis was the association between ICS use and pneumonia requiring hospitalization and any modification by bronchiectasis (nested case-control groups). However, we first measured the risk of pneumonia requiring hospitalization associated with bronchiectasis alone, after adjusting for other recognized confounders (using the main cohort). For this initial cohort analysis, we applied Cox proportional hazards for time to first pneumonia requiring hospitalization using age as the timescale variable; the assumptions of proportional hazards were met by plotting the Schoenfeld residuals and inspecting for symmetry over time.

For the main analyses of nested case-control groups 1 and 2, multivariable conditional logistic regression was used to determine effect estimates. Models were adjusted for sociodemographic variables (BMI, tobacco use status, and Index of Multiple Deprivation), COPD severity markers (Global Initiative for Chronic Obstructive Lung Disease stage, Medical Research Council dyspnea score, OCS courses, and history of exacerbations requiring hospitalization), vaccination history (pneumonia and influenza), and comorbidities (lung cancer, ILD, diabetes, cerebrovascular accident, cardiovascular disease, asthma, and depression) using complete case analysis. Interaction analyses were conducted in nested case-control group 1 for bronchiectasis and in nested case-control group 2 for BEC; likelihood ratio tests were used to test for significance and models were adjusted fully.

Several sensitivity analyses were conducted in nested case-control group 1. First, patients with OCS use in the prior year were removed if the association between ICS and CAP requiring hospitalization no longer was present or was reduced; this would suggest the association was confounded by OCS use. Second, a negative interaction analysis was conducted using a different chronic respiratory condition, ILD.^23^ This was a suitable condition because ILD is not anticipated to modify the association between ICS use and CAP requiring hospitalization; therefore, if ILD also modified the association, this would suggest residual confounding in the interaction model. Third, patients with antibiotics prescribed within 14 days before CAP requiring hospitalization occurred were removed because it could be postulated antibiotic use may explain any modification by bronchiectasis. All statistical analyses were conducted using Stata version 17 (StataCorp).

### Ethics

The protocol for this research was approved by the Independent Scientific Advisory Committee for MHRA Database Research (Identifier: 22_001718). Data management was provided by the Big Data and Analytical Unit at the Institute of Global Health Innovation.

## Results

### Characteristics of the COPD Cohort

The COPD cohort included 316,663 patients. Demographic and clinical characteristics are shown in e-Table 1. The median age was 67.9 years (interquartile range, 58.9-76.5 years) and 52.6% were male; 48.1% of the patients were using ICS at the time of cohort entry and 2.5% had bronchiectasis (e-Table 1). The COPD cohort showed a 24% increased risk of CAP requiring hospitalization associated with concomitant bronchiectasis (adjusted hazard ratio, 1.24; 95% CI, 1.15-1.33; *P* < .0001) (e-Table 2).

### Characteristics of the Nested Case-Control Groups

From the COPD cohort, 152,243 patients were eligible for the ICS new-user cohort (Fig 1, e-Table 1), from which the two nested case-control groups were drawn. The first nested case-control group contained 20,195 patients and 61,121 matched control participants. The second group was drawn from only those with COPD-bronchiectasis overlap and a recent BEC; this group contained 521 patients and 2,006 matched control participants (Fig 1, e-Table 3). Patients in nested case-control group 1 showed a mean age of 77.6 years (interquartile range, 70.0-84.0 years) and 58.4% were male. Patients in nested case-control group 2 showed a mean age of 74.3 years (interquartile range, 67.5-79.5 years) and 53.6% were male. Patients and control participants showed broadly similar BMI, comorbidities, and vaccinations, but patients showed a higher proportion with greater socioeconomic deprivation levels, more severe COPD clinical indicators, and previous CAP (Table 1, e-Table 3); however, patients in nested case control group 2 also differed in proportions of BMI categorization, some comorbidities, and higher eosinophil count.

**Table 1:** Demographic Features, Clinical Characteristics, and Comorbidities of Nested Case-Control Group 1 (With COPD)

Characteristic	Patients	Control Participants	Total
Total No.	20,195	61,121	84,316
Age, y	77.8 (70.3-84.2)	77.5 (69.9-83.9)	77.6 (70.0-84.0)
Sex			
Male	11,817 (58.5)	35,631 (58.3)	47,448 (58.4)
Female	8,378 (41.5)	25,490 (41.7)	33,868 (41.6)
Socioeconomic deprivation (IMD)			
1	2,950 (14.6)	10,059 (16.5)	13,009 (16.0)
2	3,331 (16.5)	11,130 (18.2)	14,461 (17.8)
3	3,709 (18.4)	12,047 (19.7)	15,756 (19.4)
4	4,438 (22.0)	12,761 (20.9)	17,199 (21.2)
5 (most deprived)	5,767 (28.6)	15,124 (24.7)	20,891 (25.7)
BMI			
Normal	6,553 (32.4)	17,963 (29.4)	24,516 (30.1)
Underweight	1,583 (7.8)	3,609 (5.9)	5,192 (6.4)
Overweight	7,435 (36.8)	24,266 (39.7)	31,701 (39.0)
Obese	3,834 (19.0)	12,841 (21.0)	16,675 (20.5)
Missing	790 (3.9)	2,442 (4.0)	3,232 (4.0)
COPD characteristics			
FEV_1_ % predicted			
≥ 80	2,404 (11.9)	11,012 (18.0)	13,416 (16.5)
50-79	8,533 (42.3)	27,690 (45.3)	36,223 (44.6)
30-49	5,320 (26.3)	11,638 (19.0)	16,958 (20.9)
< 30	1,298 (6.4)	2,342 (3.8)	3,640 (4.5)
Missing	2,640 (13.1)	8,439 (13.8)	11,079 (13.6)
MRC score			
1	1,391 (6.9)	7,714 (12.6)	9,105 (11.2)
2	4,076 (20.2)	16,520 (27.0)	20,596 (25.3)
3	4,869 (24.1)	13,224 (21.6)	18,093 (22.3)
4	4,603 (22.8)	8,195 (13.4)	12,798 (15.7)
5	1,924 (9.5)	2,570 (4.2)	4,494 (5.5)
Missing	3,332 (16.5)	12,898 (21.1)	16,230 (20.0)
Tobacco use history			
Never	1,099 (5.4)	4,437 (7.3)	5,536 (6.8)
Former	9,681 (47.9)	28,071 (45.9)	37,752 (46.4)
Current	8,000 (39.6)	23,790 (38.9)	31,790 (39.1)
Missing	1,415 (7.0)	4,823 (7.9)	6,238 (7.7)
Previous CAP	2,956 (14.6)	4,586 (7.5)	7,542 (9.3)
Previous LRTI	13,981 (69.2)	36,197 (59.2)	50,178 (61.7)
In year before index date			
ICS use			
Yes	10,068 (49.9)	21,376 (35.0)	31,444 (38.7)
No	10,127 (50.1)	39,745 (65.0)	49,872 (61.3)
Last ICS prescription, d			
None	10,127 (50.1)	39,745 (65.0)	49,872 (61.3)
0-30	6,079 (30.1)	12,152 (19.9)	18,231 (22.4)
30-90	2,694 (13.3)	5,741 (9.4)	8,435 (10.4)
90-180	783 (3.9)	1,925 (3.1)	2,708 (3.3)
180-365	512 (2.5)	1,558 (2.5)	2,070 (2.5)
Total ICS prescriptions			
None	10,127 (50.1)	39,745 (65.0)	49,872 (61.3)
1-3 inhalers	2,174 (10.8)	5,240 (8.6)	7,414 (9.1)
3-8 inhalers	3,835 (19.0)	8,406 (13.8)	12,241 (15.1)
9+ inhalers	4,059 (20.1)	7,730 (12.6)	11,789 (14.5)
Type of ICS			
None	10,127 (50.1)	39,745 (65.0)	49,872 (61.3)
Beclomethasone	2,055 (10.2)	5,464 (8.9)	7,519 (9.2)
Budesonide	2,078 (10.3)	5,182 (8.5)	7,260 (8.9)
Fluticasone	5,927 (29.3)	10,711 (17.5)	16,638 (20.5)
Ciclesonide/mometasone	8 (0.0)	19 (0.0)	27 (0.0)
COPD exacerbations requiring hospitalization			
0	17,038 (84.4)	58,307 (95.4)	75,345 (92.7)
1	2,165 (10.7)	2,202 (3.6)	4,367 (5.4)
2	992 (4.9)	612 (1.0)	1,604 (2.0)
OCS courses			
0	11,866 (58.8)	46,177 (75.6)	58,043 (71.4)
1	2,971 (14.7)	6,634 (10.9)	9,605 (11.8)
2	5,358 (26.5)	8,310 (13.6)	13,668 (16.8)
Comorbidities			
Bronchiectasis	984 (4.9)	1,989 (3.3)	2,973 (3.7)
Lung cancer	777 (3.8)	1,411 (2.3)	2,188 (2.7)
Asthma	3,887 (19.2)	11,156 (18.3)	15,043 (18.5)
Interstitial lung disease	668 (3.3)	1,344 (2.2)	2,012 (2.5)
Diabetes	5,022 (24.9)	14,613 (23.9)	19,635 (24.1)
CVA	2,310 (11.4)	5,824 (9.5)	8,134 (10.0)
Cardiovascular disease	14,682 (72.7)	43,076 (70.5)	57,758 (71.0)
Depression	4,292 (21.3)	11,331 (18.5)	15,623 (19.2)
Vaccinations			
Pneumonia vaccine (ever)	2,380 (11.8)	6,880 (11.3)	9,260 (11.4)
Influenza vaccine (prior year)	3,931 (19.5)	12,367 (20.2)	16,298 (20.0)

Data are presented as No. (%) or median (interquartile range). CAP = community-acquired pneumonia; CVA = cerebrovascular accident; ICS = inhaled corticosteroids; IMD = Index of Multiple Deprivation; LRTI = lower respiratory tract infection; MRC = Medical Research Council; OCS = oral corticosteroids.

### Association Between ICS and Pneumonia

A 26% increased odds of CAP requiring hospitalization was associated with using ICS (adjusted OR [AOR], 1.26; 95% CI, 1.19-1.32) (Table 2). Recent ICS use showed the strongest association, but the association was no longer significant by 180 days after ICS use (0-30 days: AOR, 1.27; 95% CI, 1.20-1.35; 30-90 days: AOR, 1.32; 95% CI, 1.22-1.42; 90-180 days: AOR, 1.14; 95% CI, 1.00-1.29; 180-365 days: AOR, 1.05; 95% CI, 0.90-1.22) (Table 2). The cumulative number of ICS prescriptions in the year before the index date did not affect the strength of the association (Table 2). Assessing the effect of different ICS drugs, we found an increased odds with fluticasone (AOR, 1.42; 95% CI, 1.33-1.51) and beclomethasone (AOR, 1.15; 95% CI, 1.06-1.25), but not with budesonide (AOR, 1.04; 95% CI, 0.95-1.12).

**Table 2:** Association Between Community-Acquired Pneumonia and ICS, Time Since Last ICS Prescription, No. of ICS Prescriptions, and Type of ICS Drug in Nested Case-Control Group 1 (With COPD; n = 51,335)

Variable	Adjusted OR	*P* Value	95% CI
ICS use in past year			
No	...	Reference	...
Yes	1.26	< .001	1.19-1.32
Time since last ICS, d			
None	...	Reference	...
0-30	1.27	< .001	1.20-1.35
30-90	1.32	< .001	1.22-1.42
90-180	1.14	> .05	1.00-1.29
180-365	1.05	.530	0.90-1.22
No. of ICS inhalers in past year			
None	...	Reference	...
1-3	1.26	< .001	1.16-1.37
4-8	1.25	< .001	1.17-1.34
≥ 9	1.25	< .001	1.17-1.34
Type of ICS used in past year			
None	...	Reference	...
Beclomethasone	1.15	< .01	1.06-1.25
Budesonide	1.04	.377	0.95-1.12
Fluticasone	1.42	< .001	1.33-1.51
Ciclesonide/mometasone	0.55	.364	0.15-1.98

Adjusted for socioeconomic deprivation (Index of Multiple Deprivation), BMI, FEV_1_ % predicted, Medical Research Council dyspnea score, tobacco use history, history of pneumonia, history of lower respiratory tract infections, hospital exacerbations in the past year, courses of oral corticosteroids in the past year, bronchiectasis, lung cancer, asthma, interstitial lung disease, diabetes, cerebrovascular accidents, depression, pneumonia vaccine (ever), and influenza vaccine (year prior). ICS = inhaled corticosteroids.

### Association of ICS With CAP Requiring Hospitalization in COPD With Concomitant Bronchiectasis

In nested case-control group 1, concomitant bronchiectasis was found to be a significant modifier of the association between ICS and CAP requiring hospitalization (*P* < .01, likelihood ratio test). In patients with COPD-bronchiectasis overlap, ICS was not associated significantly with pneumonia (AOR, 1.01; 95% CI, 0.8-1.28), unlike in COPD without bronchiectasis (AOR, 1.27; 95% CI, 1.20-1.34). In the negative exposure analysis, ILD was not found to be an effect modifier, suggesting that significant residual confounding in the model was unlikely (*P* > .05, likelihood ratio test).

Two other sensitivity analyses were applied. First, removing patients with OCS use in the previous year had a negligible impact on the effect estimates (e-Table 4). Second, assessing the influence of recent antibiotic use, antibiotic use was prevalent in all patients, especially those with bronchiectasis: 70.8% of bronchiectasis patients had been prescribed antibiotics in the year prior, although the use of macrolides occurred in only around 2% of patients (e-Table 5). Excluding patients with antibiotic prescription in the 14 days before the index date had a negligible impact on the effect estimates (e-Table 6). As a further sensitivity analysis, nested case-control group 2 (containing only patients with COPD-bronchiectasis overlap) was drawn. Again, ICS use was not associated with CAP requiring hospitalization in those with COPD-bronchiectasis overlap (AOR, 1.05; 95% CI, 0.78-1.41; *P* = .75).

### The Association With BEC, ICS Use, and CAP Requiring Hospitalization in COPD-Bronchiectasis Overlap

Nested case-control group 2 also was used to investigate any modification effect from BEC. An interaction analysis found eosinophils were a significant modifier of the association between ICS use and CAP requiring hospitalization (*P* < .05, likelihood ratio test). High BEC (> 0.3 × 10^9^/L) was not associated with CAP requiring hospitalization (AOR, 0.89; 95% CI, 0.53-1.24), but a normal eosinophil count (≤ 0.3 × 10^9^/L) was associated with CAP requiring hospitalization (AOR, 1.56; 95% CI, 0.78-1.41).

## Discussion

In this study, we identified a cohort of > 300,000 patients with COPD and investigated the association of ICS use and pneumonia requiring hospitalization in those with concomitant bronchiectasis. Using a cohort design, we initially determined that concomitant bronchiectasis was associated with a 24% increased risk of CAP requiring hospitalization, replicating findings from one earlier observational study in US data and landmark trials.^24^, ^25^, ^26^ Next, we used a nested case-control design to determine that ICS use was associated with 26% increased odds of CAP requiring hospitalization and that this occurred in a time-dependent manner. Finally, by using an interaction analysis, we were able to determine that in people with COPD-bronchiectasis overlap, ICS use did not augment further the already elevated bronchiectasis-associated pneumonia risk. All models were adjusted for multiple potential confounders including COPD severity and vaccination status. Furthermore, to explore if additional residual confounding was present, we conducted a negative control exposure analysis. The negative exposure chosen was another chronic respiratory condition, ILD, that would not be expected to modify the association between ICS use and pneumonia. Therefore, if ILD was found also to modify the association, this would imply that the case-control model was confounded; however, ILD did not modify the association. Another sensitivity analysis was to draw a second nested case-control group, but this time containing only those with COPD-bronchiectasis overlap, resulting in a much smaller sample size. Again, we found the same effect, that ICS use did not further increase the already elevated pneumonia risk. Other sensitivity analyses conducted, including removing patients with antibiotic prescriptions or OCS use, also did not change the effect estimate.

Our data demonstrated an ICS association with CAP that is dependent on the time since last ICS use, but no association with the total number of ICS inhalers used. These findings imply that pneumonia risk relates to the proximity of ICS use, rather than the cumulative effect of ICS exposure. Postulated biological mechanisms include ICS-induced alterations of the innate and adaptive immune system,^27^^,^^28^ which may increase bacterial load and change the microbial composition in the airways.^29^ The odds of pneumonia were increased 1.42-fold with fluticasone and 1.15-fold with beclomethasone, but were not increased with budesonide, which enters the lungs with a lower lipophilicity and dissolves more quickly into pulmonary fluids, leading to a reduced local effect because of a more rapid passage into the systemic circulation,^30^ supporting findings in the Canadian observational study by Suissa et al.^31^

People with bronchiectasis may receive antibiotics for respiratory infections earlier than those without bronchiectasis. Additionally, they may receive long-term prophylactic macrolides.^18^ Differential early use of antibiotics potentially could have explained the relatively reduced risk of ICS-associated pneumonia in those with bronchiectasis. Indeed, in our cohort, those with bronchiectasis were more likely to receive antibiotics, but the overall use of macrolides was very low in this primary care population. Furthermore, excluding patients prescribed antibiotics had negligible consequences on the overall effect estimates.

Neutrophils are the most abundant airway inflammatory cell in bronchiectasis, but the importance of eosinophils recently was recognized.^32^ In a post hoc analysis of bronchiectasis trial data from 86 participants, inhaled fluticasone reduced bronchiectasis exacerbations in those with BEC of ≥ 150 cells/μL.^33^ A large European multicenter cohort of 1,007 patients with bronchiectasis reported that approximately one-fifth of patients exhibited sputum eosinophil counts of > 3%.^34^ They showed that elevated BECs (> 300 cells/μL) were an effective surrogate marker of sputum eosinophils, and these were associated with infective exacerbations, suggesting that in bronchiectasis, eosinophilic inflammation and microbial dysbiosis are interrelated. In contrast, in COPD, a lack of eosinophilic inflammation is associated with chronic bacterial infection. Martinez-Garcia et al^17^ demonstrated in a microbiology study of 201 carefully characterized patients with COPD (57% also had bronchiectasis) that those with low BECs had an increased incidence of bacterial colonization and pneumonia and that ICS use further increased the risk. A pooled analysis of 10 randomized control trials of ICS-containing combination treatment in patients with COPD found that the risk of pneumonia was 31% higher in patients with a BEC of < 2% vs ≥ 2%.^35^ Additionally, using real-world observational data, patients with COPD using ICS-containing inhalers with a BEC of < 150 cells/μL again exhibited a higher risk of pneumonia.^36^ Correspondingly, the authors of the Global Initiative for Chronic Obstructive Lung Disease Science Committee recently published a review article that concludes that low BECs are associated with an increased risk of recurrent bacterial infections and pneumonia in COPD and that these risks likely are increased by ICS use.^22^ Therefore, our study findings parallel these earlier studies showing that low BEC is associated with higher pneumonia risk, albeit using a different methodologic approach, and ours is the first study to study this directly in a population with COPD-bronchiectasis overlap. Furthermore, a recent publication from a large Spanish bronchiectasis registry found that ICS use reduces the risk of a bronchiectasis exacerbation only in those with elevated BEC of > 300 cells/μL.^37^ Overall, these findings suggest that ICS may be beneficial with low risk of pneumonia in patients with elevated BEC and COPD-bronchiectasis overlap, in keeping with the recent recommendations in the European Respiratory Society adult bronchiectasis guidelines. However, other potential side effects from ICS use should be considered.

### Study Limitations

This observational study has several limitations. First, as with all routinely collected database studies, we did not have information on patient adherence to the ICS prescriptions. This may have biased effect estimates towards null. Second, the diagnosis of bronchiectasis requires radiologic imaging, a limited resource that likely led to underdiagnosis in this cohort, a well-recognized problem in bronchiectasis prevalence studies.^11^^,^^12^ In addition, the increase of participants who did not use tobacco in the case-control groups may suggest that several of these patients in fact had primary bronchiectasis with airflow obstruction, but were labelled as having comorbid COPD, which could impact on the effect of ICS. Third, we did not have access to microbiology data or data on severity of bronchiectasis for each patient. Fourth, we did not have information on pneumonia risk factors, alcohol use (this is often not recorded and at risk of misclassification), or neurologic conditions managed in secondary care that may cause oropharyngeal dysphagia. Fifth, we assessed only for increase in pneumonia requiring hospitalization and did not assess nonpneumonia requiring hospitalization, which many of the randomized controlled trials in COPD reported as increasing.^6^ Finally, although biological plausibility exists and we have endeavored to identify and minimize bias, we have not made causal statements because of the observational nature of the study.

## Interpretation

Our main finding is that although ICS use and concomitant bronchiectasis independently increase the risk of pneumonia requiring hospitalization, no further augmentation from ICS use occurs in patients with COPD-bronchiectasis overlap. Additionally, in our smaller cohort of only those with COPD-bronchiectasis overlap, we found that an elevated BEC seems to protect patients from the ICS-associated increased pneumonia risk, reflecting findings found in previous cohorts with COPD.

## Funding/Support

Supported by the 10.13039/501100000272National Institute for Health Research Imperial Biomedical Research Centre.

## Financial/Nonfinancial Disclosures

None declared.

## Supplementary Data

e-Online Data
