# Corrigendum to: *CHEST*. 2023;164(4):875-884

**DOI:** 10.1016/j.chest.2024.01.041

**Published:** 2024-03-07

The October 2023 original research article titled “The Effect of Inhaled Corticosteroids on Pneumonia Risk in Patients With COPD-Bronchiectasis Overlap: A UK Population-Based Case-Control Study” by Ritchie et al (*CHEST*. 2023; 164[4]:875-884) contained the following error: the surname of author Aran Singanayagam was misspelled in the text.

The online version of this article and its supplementary content have been corrected.

