# Supplementary material for: The Effect of Inhaled Corticosteroids on Pneumonia Risk in Patients With COPD-Bronchiectasis Overlap: A UK Population-Based Case-Control Study
Source: Chest. 2023 Jun 17;164(4):875–84. doi: 10.1016/j.chest.2023.06.007 (PMC10808068; doi:10.1016/j.chest.2023.06.007)
Supplement: e-Online Data [file mmc1.docx]

e-Table 1. Demographics and characteristics of the COPD cohort and by their inclusion or not in the ICS new-user cohort.

| **COPD COHORT** | **ICS new-user cohort** | | **Total COPD cohort** |
| --- | --- | --- | --- |
|  | **Yes** | **No** |  |
| **Total number** | 152243 (48.1%) | 164420 (51.9%) | 316663 |
| **Age (years), median** | 67.5  (IQR 58.9-76.0) | 68.4  (IQR 59.0-76.6) | 67.9  (IQR 58.9-76.5) |
| **Gender** |  |  |  |
| Male | 85468 (56.1%) | 81047 (49.3%) | 166515 (52.6%) |
| Female | 66775 (43.9%) | 83373 (50.7%) | 150148 (47.4%) |
| **Socioeconomic deprivation (IMD)** |  |  |  |
| 1 | 22671 (14.9%) | 25415 (15.5%) | 48086 (15.2%) |
| 2 | 26650 (17.5%) | 28231 (17.2%) | 54881 (17.3%) |
| 3 | 29594 (19.4%) | 31226 (19.0%) | 60820 (19.2%) |
| 4 | 33046 (21.7%) | 35465 (21.6%) | 68511 (21.6%) |
| 5 (most deprived) | 40179 (26.4%) | 43956 (26.7%) | 84135 (26.6%) |
| Missing | 103 (0.1%) | 127 (0.1%) | 230 (0.1%) |
| **BMI** |  |  |  |
| Normal | 46330 (30.4%) | 45894 (27.9%) | 92224 (29.1%) |
| Underweight | 6810 (4.5%) | 6597 (4.0%) | 13407 (4.2%) |
| Overweight | 59480 (39.1%) | 63670 (38.7%) | 123150 (38.9%) |
| Obese | 33911 (22.3%) | 40748 (24.8%) | 74659 (23.6%) |
| Missing | 5712 (3.8%) | 7511 (4.6%) | 13223 (4.2%) |
| **COPD characteristics** |  |  |  |
| **FEV_1_ % predicted** |  |  |  |
| ≥80% | 27836 (18.3%) | 23423 (14.2%) | 51259 (16.2%) |
| 50-79% | 68733 (45.1%) | 66000 (40.1%) | 134733 (42.5%) |
| 30-49% | 28579 (18.8%) | 37298 (22.7%) | 65877 (20.8%) |
| <30% | 6145 (4%) | 11492 (7%) | 17637 (5.6%) |
| Missing | 20950 (13.8%) | 26207 (15.9%) | 47157 (14.9%) |
| **MRC score** |  |  |  |
| 1 | 21677 (14.2%) | 16013 (9.7%) | 37690 (11.9%) |
| 2 | 41682 (27.4%) | 36924 (22.5%) | 78606 (24.8%) |
| 3 | 29690 (19.5%) | 34060 (20.7%) | 63750 (20.1%) |
| 4 | 17950 (11.8%) | 26770 (16.3%) | 44720 (14.1%) |
| 5 | 5506 (3.6%) | 10211 (6.2%) | 15717 (5.0%) |
| Missing | 35738 (23.5%) | 40442 (24.6%) | 76180 (24.1%) |
| **Smoking history** |  |  |  |
| Never | 4934 (3.2%) | 6247 (3.8%) | 11181 (3.5%) |
| Ex-smoker | 83805 (55.0%) | 101075 (61.5%) | 184880 (58.4%) |
| Current | 62020 (40.7%) | 55800 (33.9%) | 117820 (37.2%) |
| Missing | 1484 (1.0%) | 1298 (0.8%) | 2782 (0.9%) |
| **Hospitalised COPD exacerbations** |  |  |  |
| 0 | 147035 (96.6%) | 152940 (93.0%) | 299975 (94.7%) |
| ≥1 | 5208 (3.4%) | 11480 (7.0%) | 16688 (5.3%) |
| **OCS courses** |  |  |  |
| 0 | 128464 (84.4%) | 99442 (60.5%) | 227906 (72.0%) |
| 1 | 14843 (9.7%) | 27314 (16.6%) | 42157 (13.3%) |
| 2 | 8936 (5.9%) | 37664 (22.9%) | 46600 (14.7%) |
| **Comorbidities** |  |  |  |
| **Bronchiectasis** | 2687 (1.8%) | 5211 (3.2%) | 7898 (2.5%) |
| **Previous CAP** | 7730 (5.1%) | 11780 (7.2%) | 19510 (6.2%) |
| **Previous LRTI** | 71508 (47.0%) | 90993 (55.3%) | 162501 (51.3%) |
| **Lung cancer** | 1135 (0.7%) | 1069 (0.7%) | 2204 (0.7%) |
| **Asthma** | 6859 (4.5%) | 64506 (39.2%) | 71365 (22.5%) |
| **Interstitial lung disease** | 2033 (1.3%) | 2395 (1.5%) | 4428 (1.4%) |
| **Diabetes** | 19173 (12.6%) | 21578 (13.1%) | 40751 (12.9%) |
| **CVA** | 7811 (5.1%) | 8230 (5.0%) | 16041 (5.1%) |
| **Cardiovascular disease** | 58711 (38.6%) | 63025 (38.3%) | 121736 (38.4%) |
| **Depression** | 23697 (15.6%) | 27943 (17.0%) | 51640 (16.3%) |

e-Table 2. Association between hospitalized pneumonia and bronchiectasis in the COPD cohort.

|  | **Adjusted HR** | **p-value** | **95% CI** |
| --- | --- | --- | --- |
| **Bronchiectasis** | 1.24 | <0.0001 | 1.15-1.33 |
| **Gender** |  |  |  |
| Male | *Reference* | | |
| Female | 0.91 | <0.0001 | 0.89-0.93 |
| **Socioeconomic deprivation (IMD)** |  |  |  |
| 1 | *Reference* | | |
| 2 | 1.01 | 0.63 | 0.97-1.05 |
| 3 | 1.01 | 0.69 | 0.97-1.05 |
| 4 | 1.01 | 0.73 | 0.97-1.05 |
| 5 (most deprived) | 1.07 | <0.0001 | 1.03-1.11 |
| **BMI** |  |  |  |
| Normal | *Reference* | | |
| Underweight | 1.28 | <0.0001 | 1.21-1.35 |
| Overweight | 0.83 | <0.0001 | 0.81-0.85 |
| Obese | 0.70 | <0.0001 | 0.68-0.72 |
| **Previous CAP** | 1.46 | <0.0001 | 1.4-1.52 |
| **Previous LRTI** | 1.09 | <0.0001 | 1.07-1.12 |
| **MRC score** |  |  |  |
| 1 | *Reference* | | |
| 2 | 1.46 | <0.0001 | 1.39-1.54 |
| 3 | 2.29 | <0.0001 | 2.18-2.41 |
| 4 | 3.22 | <0.0001 | 3.06-3.39 |
| 5 | 4.47 | <0.0001 | 4.22-4.73 |
| **FEV_1_ % predicted** |  |  |  |
| ≥80% | *Reference* | | |
| 50-79% | 1.34 | <0.0001 | 1.29-1.4 |
| 30-49% | 1.94 | <0.0001 | 1.85-2.02 |
| <30% | 2.21 | <0.0001 | 2.09-2.33 |
| **Hospitalised COPD exacerbations** |  |  |  |
| 0 | *Reference* | | |
| ≥1 | 1.73 | <0.0001 | 1.65-1.81 |
| **OCS courses** |  |  |  |
| 0 | *Reference* | | |
| 1 | 1.01 | 0.43 | 0.98-1.05 |
| 2 | 1.21 | <0.0001 | 1.17-1.25 |
| **Smoking history** | *Reference* | | |
| Never | 1.17 | <0.001 | 1.06-1.28 |
| Ex-smoker | 1.17 | <0.001 | 1.06-1.28 |
| Current | 0.96 | 0.39 | 0.87-1.05 |
| **Diabetes** | 1.21 | <0.0001 | 1.17-1.25 |
| **Cardiovascular disease** | 1.33 | <0.0001 | 1.3-1.37 |
| **CVA** | 1.40 | <0.0001 | 1.33-1.47 |
| **Lung cancer** | 1.90 | <0.0001 | 1.63-2.22 |
| **Interstitial lung disease** | 1.63 | <0.0001 | 1.49-1.78 |

* Adjusted for all covariates shown in the table; CVA=cerebrovascular accident, CAP = community acquire pneumonia, LRTI=lower respiratory tract infection; OCS=oral corticosteroids; COPD=chronic obstructive pulmonary disease; HR=hazard ratio; CI=confidence interval. N=316,663.

e-Table 3. Demographics and characteristics of nested case-control 2 (COPD and concomitant bronchiectasis)

| **NESTED CASE CONTROL 2** | **Cases** | **Controls** | **Total** |
| --- | --- | --- | --- |
| **Total number** | 521 | 2,006 | 2,527 |
| **Age (years), median** | 74.4 | 74.3 | 74.3 |
|  | (67.6-79.5) | (67.5-79.5) | (67.5-79.5) |
| **Gender** **(males)** | 281 (53.9%) | 1074 (53.5%) | 1355 (53.6%) |
| **Socioeconomic deprivation (IMD)** |  |  |  |
| 1 | 90 (17.3%) | 449 (22.4%) | 539 (21.3%) |
| 2 | 103 (19.8%) | 418 (20.8%) | 521 (20.6%) |
| 3 | 115 (22.1%) | 421 (21.0%) | 536 (21.2%) |
| 4 | 101 (19.4%) | 377 (18.8%) | 478 (18.9%) |
| 5 (most deprived) | 112 (21.5%) | 340 (16.9%) | 452 (17.9%) |
| **BMI** |  |  |  |
| Normal | 189 (36.3%) | 616 (30.7%) | 805 (31.9%) |
| Underweight | 47 (9.0%) | 116 (5.8%) | 163 (6.5%) |
| Overweight | 199 (38.2%) | 866 (43.2%) | 1065 (42.1%) |
| Obese | 77 (14.8%) | 381 (19.0%) | 458 (18.1%) |
| Missing | 9 (1.7%) | 27 (1.3%) | 36 (1.4%) |
| **FEV_1_ % predicted** |  |  |  |
| ≥80% | 117 (22.5%) | 633 (31.6%) | 750 (29.7%) |
| 50-79% | 191 (36.7%) | 723 (36.0%) | 914 (36.2%) |
| 30-49% | 89 (17.1%) | 288 (14.4%) | 377 (14.9%) |
| <30% | 19 (3.6%) | 32 (1.6%) | 51 (2.0%) |
| Missing | 105 (20.2%) | 330 (16.5%) | 435 (17.2%) |
| **MRC score** |  |  |  |
| 1 | 52 (10.0%) | 320 (16.0%) | 372 (14.7%) |
| 2 | 135 (25.9%) | 653 (32.6%) | 788 (31.2%) |
| 3 | 128 (24.6%) | 396 (19.7%) | 524 (20.7%) |
| 4 | 81 (15.5%) | 201 (10.0%) | 282 (11.2%) |
| 5 | 18 (3.5%) | 44 (2.2%) | 62 (2.5%) |
| Missing | 107 (20.5%) | 392 (19.5%) | 499 (19.7%) |
| **Smoking history** |  |  |  |
| Never | 82 (15.7%) | 347 (17.3%) | 429 (17.0%) |
| Ex-smoker | 264 (50.7%) | 939 (46.8%) | 1203 (47.6%) |
| Current | 113 (21.7%) | 409 (20.4%) | 522 (20.7%) |
| Missing | 62 (11.9%) | 311 (15.5%) | 373 (14.8%) |
| **Blood eosinophil count x10^9^/L** |  |  |  |
| Median (IQR) | 0.30 (0.20-0.40) | 0.26 (0.19-0.04) | 0.27 (0.19-0.40) |
| High | 276 (53.0%) | 736 (36.7%) | 954 (37.8%) |
| Normal | 218 (41.8%) | 1126 (56.1%) | 1402 (55.5%) |
| Missing | 27 (5.2%) | 144 (7.2%) | 171 (6.8%) |
| **Previous CAP** | 172 (33.0%) | 390 (19.4%) | 562 (22.2%) |
| **Previous LRTI** | 433 (83.1%) | 1492 (74.4%) | 1925 (76.2%) |
| **ICS use** |  |  |  |
| Yes | 392 (75.2%) | 1626 (81.1%) | 2018 (79.9%) |
| No | 129 (24.8%) | 380 (18.9%) | 509 (20.1%) |
| **OCS courses** |  |  |  |
| 0 | 297 (57.0%) | 1489 (74.2%) | 1786 (70.7%) |
| 1 | 73 (14.0%) | 212 (10.6%) | 285 (11.3%) |
| 2 | 151 (29.0%) | 305 (15.2%) | 456 (18.0%) |
| **Lung cancer** | 14 (2.7%) | 54 (2.7%) | 68 (2.7%) |
| **Asthma** | 133 (25.5%) | 516 (25.7%) | 649 (25.7%) |
| **Interstitial lung disease** | 36 (6.9%) | 103 (5.1%) | 139 (5.5%) |
| **Diabetes** | 165 (31.7%) | 506 (25.2%) | 671 (26.6%) |
| **Cerebrovascular accident** | 65 (12.5%) | 163 (8.1%) | 228 (9.0%) |
| **Cardiovascular disease** | 357 (68.5%) | 1430 (71.3%) | 1787 (70.7%) |
| **Depression** | 134 (25.7%) | 388 (19.3%) | 522 (20.7%) |
| **Pneumonia vaccine** | 74 (14.2%) | 328 (16.4%) | 402 (15.9%) |

e-Table 4. Association between community acquired pneumonia and time since last ICS prescription in patients without use of OCS in year prior

| **NESTED CASE CONTROL 1** | **Adjusted OR** | **p-value** | **95% CI** |
| --- | --- | --- | --- |
| **Time since last ICS** |  |  |  |
| No ICS | *Reference* | | |
| 0-30 days | 1.42 | <0.001 | 1.34-1.52 |
| 30-90 days | 1.40 | <0.001 | 1.30-1.52 |
| 90-180 days | 1.17 | 0.030 | 1.01-1.34 |
| 180-365 days | 1.10 | 0.248 | 0.94-1.29 |

* Adjusted for socioeconomic deprivation (IMD), body mass index, FEV_1_ percent predicted, MRC dyspnoea score, smoking history, history of pneumonia, lower respiratory tract infections, hospital exacerbations in the past year, bronchiectasis, lung cancer, asthma, interstitial lung disease, diabetes mellitus, cerebrovascular accidents, depression, pneumonia vaccine (ever) and influenza vaccine (year prior). ICS = inhaled corticosteroids.

e-Table 5. Antibiotic prescriptions in the cases and controls, comparing those COPD patients with concomitant bronchiectasis and those without.

|  | **No bronchiectasis** | | **Bronchiectasis** | |
| --- | --- | --- | --- | --- |
| **NESTED CASE CONTROL 1** | **N** | **%** | **N** | **%** |
| **Antibiotics in year prior** | 43,868 | 56.0 | 2,106 | 70.8 |
| **Macrolides in year prior** | 218 | 0.3 | 62 | 2.1 |
| **Antibiotics in 14 days pre-CAP** | 6,993 | 8.9 | 419 | 14.1 |
| **TOTAL** | 78,343 | 100.0 | 2,973 | 100.0 |

Binary variables, treating the patient as contributing to the total N if the patient had at least one prescription in the time period specified.

e-Table 6. Interaction analysis with bronchiectasis as effect modifier for the association between ICS and community acquired pneumonia in COPD patients that have not used antibiotics within 14 days prior to the hospitalised pneumonia

| **NESTED CASE CONTROL 1** | **Adjusted OR** | **p-value** | **95% CI** |
| --- | --- | --- | --- |
| ***Concomitant bronchiectasis*** | |  |  |
| **ICS in past year** |  |  |  |
| No | *Reference* | | |
| Yes | 0.86 | 0.245 | 0.67-1.11 |
| ***No bronchiectasis*** |  |  |  |
| **ICS in past year** |  |  |  |
| No | *Reference* | | |
| Yes | 1.28 | <0.001 | 1.21-1.36 |

* Adjusted for socioeconomic deprivation (IMD), body mass index, FEV_1_ percent predicted, MRC dyspnoea score, smoking history, history of pneumonia, lower respiratory tract infections, hospital exacerbations in the past year, courses of oral corticosteroids in the past year, bronchiectasis, lung cancer, asthma, interstitial lung disease, diabetes mellitus, cerebrovascular accidents, depression, pneumonia vaccine (ever) and influenza vaccine (year prior). ICS = inhaled corticosteroids.
